# How to integrate monoclonal antibodies targeting the calcitonin gene-related peptide or its receptor in daily clinical practice

**DOI:** 10.1186/s10194-019-1000-5

**Published:** 2019-05-06

**Authors:** Cindy Tiseo, Raffaele Ornello, Francesca Pistoia, Simona Sacco

**Affiliations:** Neuroscience Section, Department of Applied Clinical Sciences and Biotechnology, University of L’Aquila, 67100 L’Aquila, Italy

**Keywords:** Migraine, Calcitonin gene-related peptide, Preventive treatment, Monoclonal antibodies

## Abstract

**Background:**

Migraine is a major public health issue associated with significant morbidity, considerable negative impact on quality of life, and significant socioeconomic burden. Preventive treatments are required to reduce the occurrence and the severity of acute attacks and to minimize the use of abortive medications and the associate risk of drug-related adverse events, as well as the onset of medication-overuse headache and chronification of migraine. We performed a review of all available evidence on the safety and efficacy of monoclonal antibodies targeting the calcitonin gene-related peptide or its receptor for the preventive treatment of migraine to provide evidence-based guidance on their use in clinical practice.

**Abstract main body:**

Monoclonal antibodies targeting the calcitonin gene-related peptide or its receptor are mechanism-specific drugs for the preventive treatment of migraine. Double-blind randomized clinical trials have shown that monoclonal antibodies targeting the calcitonin gene-related peptide or its receptor are effective across all the spectrum of migraine patients who require prevention and have a good safety and tolerability profile. Nevertheless, high costs limit the affordability of those drugs at the moment.

**Conclusions:**

Specificity, long half-life, efficacy, tolerability, and ease of use make monoclonal antibodies targeting the calcitonin gene-related peptide or its receptor an appealing treatment option for migraine prevention. Optimal strategies to manage treatment over time still need to be clarified with real-life data.

## Introduction

Migraine is a chronic neurologic disease affecting around 15% of adult subjects, with a higher female prevalence [1–3]. It is a major public health issue associated with significant morbidity, considerable negative impact on quality of life, and considerable socioeconomic burden [4]. Patients with migraine attacks recurring at high frequency, or being associated with pain of severe intensity, or determining a reduction of quality of life require preventive treatments. In recent years, advances in the understanding of migraine pathophysiology paved the way for the development of migraine-specific preventive treatments. Early after the discovery of calcitonin-gene related peptide (CGRP) [5, 6], it was clear that it would have a crucial role in the pathophysiology of migraine [7–12]. CGRP is a neuropeptide existing in 2 isoforms, α and β. The α isoform, which is primarily implied in the pathogenesis of migraine pain, is a 37-aminoacid peptide synthesized by peripheral sensory neurons and by numerous sites in the central nervous system through alternative splicing of the calcitonin gene mRNA [5]. The β isoform is encoded from a different gene and it is expressed primarily by the enteric sensory system [8]. The receptors for members of the CGRP peptide family consist of two G protein-coupled receptors, the calcitonin-like receptor (CLR; a seven-transmembrane receptor component) and the receptor component protein (RCP), interacting with the receptor activity-modifying protein 1 (RAMP1). CGRP receptor is expressed by the trigeminal neurons, the smooth muscle cells of peripheral intracranial vasculature, the dura mater, and the brainstem [8].

Experimental studies revealed that CGRP levels are increased during the migraine attack [9] and tend to normalize together with pain relief [10]. Interestingly, the intravenous administration of CGRP can induce migraine-like headache in migraineurs but not in healthy subjects [13]. The current hypothesis on migraine pathogenesis suggests that migraine initiates in the brain with cortical and subcortical changes inducing the activation of the trigeminovascular system with subsequent transmission of pain signals to the thalamus [14]. The activation of trigeminovascular system seems a crucial step for the complete expression of migraine attack and its accompanying symptoms [15]. Following the activation of the trigeminovascular system, CGRP is released at trigeminal endings and induces vasodilation of the intracranial arteries, modulates neuronal excitability through the facilitation of pain transmission, and activates neurogenic inflammation.

Based on all those findings, CGRP gained importance as a potential pharmacological target for migraine prevention. Small molecules acting as competitive CGRP receptor antagonists, the gepants, have been proven effective in the acute and prophylactic treatment of migraine attacks, but their clinical development was limited by safety concerns regarding liver toxicity following continuous exposure to the initial drugs [16]. Therefore, the attention shifted to the development of monoclonal antibodies (MoAbs) targeting CGRP or its receptor. The mechanism of action of MoAbs in migraine treatment includes the modulation of CGRP-induced pain transmission and the reduction of both peripheral and central sensitization through the removal of the excess of released CGRP (CGRP MoAbs) or the block of the ligand from binding the CGRP receptor (CGRP receptor MoAbs) [17].

We performed a review of all available evidence on the safety and efficacy of CGRP(r) MoAbs in patients with migraine to provide evidence-based guidance on their use in clinical practice.

## Methods

We searched papers indexed in PubMed and Scopus from inception to December 2018 using the following search terms: “migraine OR headache AND (CGRP OR eptinezumab OR galcanezumab OR fremanezumab OR erenumab)”. We also performed a manual search among contributions presented to the main headache conferences including the European Headache Federation, the International Headache Conference, and the American Academy of Neurology during the years 2017 and 2018. A manual search among references lists and Google Scholar citations of selected articles and reviews was also performed. Only studies published in English were considered.

We included data from phase III clinical trials, or phase II trials if phase III trials were lacking, which tested the CGRP(r) MoAbs doses to be used in clinical practice.

## Available evidence on efficacy

To date, four IgG MoAbs acting on the CGRP pathway have been developed and tested in humans: eptinezumab, erenumab, fremanezumab and galcanezumab [18–31]. Erenumab is a fully human MoAb, binding the CGRP receptor, while eptinezumab, fremanezumab, and galcanezumab are fully humanized MoAbs binding CGRP.

Eptinezumab has been studied in a phase II randomized clinical trial (RCT) [18] for the prevention of episodic migraine (EM) at the dose of 1000 mg. Ongoing phase III RCTs are testing the quarterly intravenous administration of eptinezumab 30 mg, 100 mg and 300 mg for the prevention of frequent EM (PROMISE-1) [19–21], and of eptinezumab 100 mg and 300 mg for chronic migraine (CM) (PROMISE-2) [22, 23]. Erenumab has been studied in phase III RCTs (STRIVE and ARISE) for the prevention of EM [24, 25] and in phase II RCT [26] for the prevention of CM at the doses of 70 mg and 140 mg administered subcutaneously monthly. Fremanezumab has been investigated in phase III RCTs at the doses of 225 mg administered monthly and 675 mg quarterly for the prevention of EM (HALO-EM) [27], and at the doses of 675 mg quarterly and of 225 mg monthly (with a single loading dose of 675 mg) for the prevention of CM (HALO-CM) [28]. Galcanezumab has been studied in phase III RCTs at the doses of 120 mg (with a single loading dose of 240 mg) and of 240 mg administered monthly for the prevention of EM (EVOLVE-1 and EVOLVE-2) [29, 30] and CM (REGAIN) [31].

**Fig. 1:**
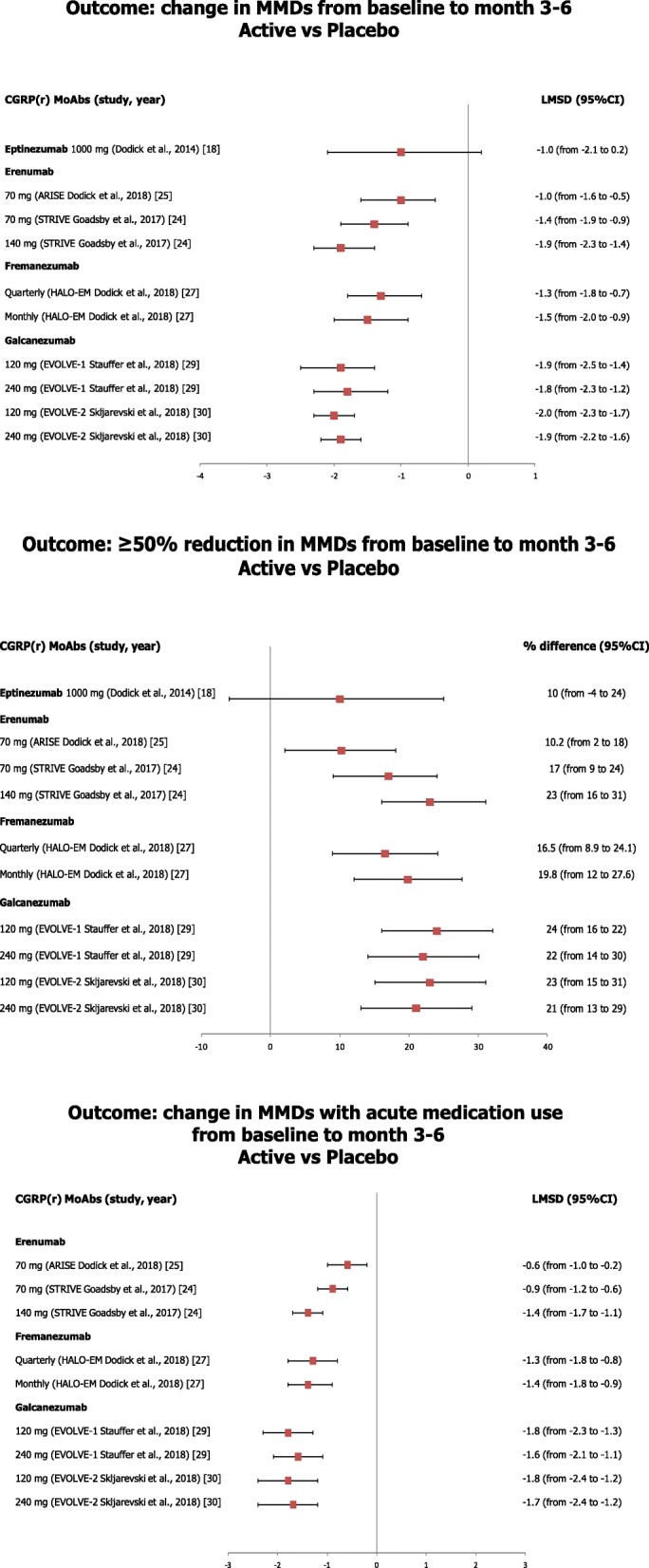
Data showing the results of treatment with CGRP(r) MoAbs on the main efficacy endpoints in patients with episodic migraine

**Fig. 2:**
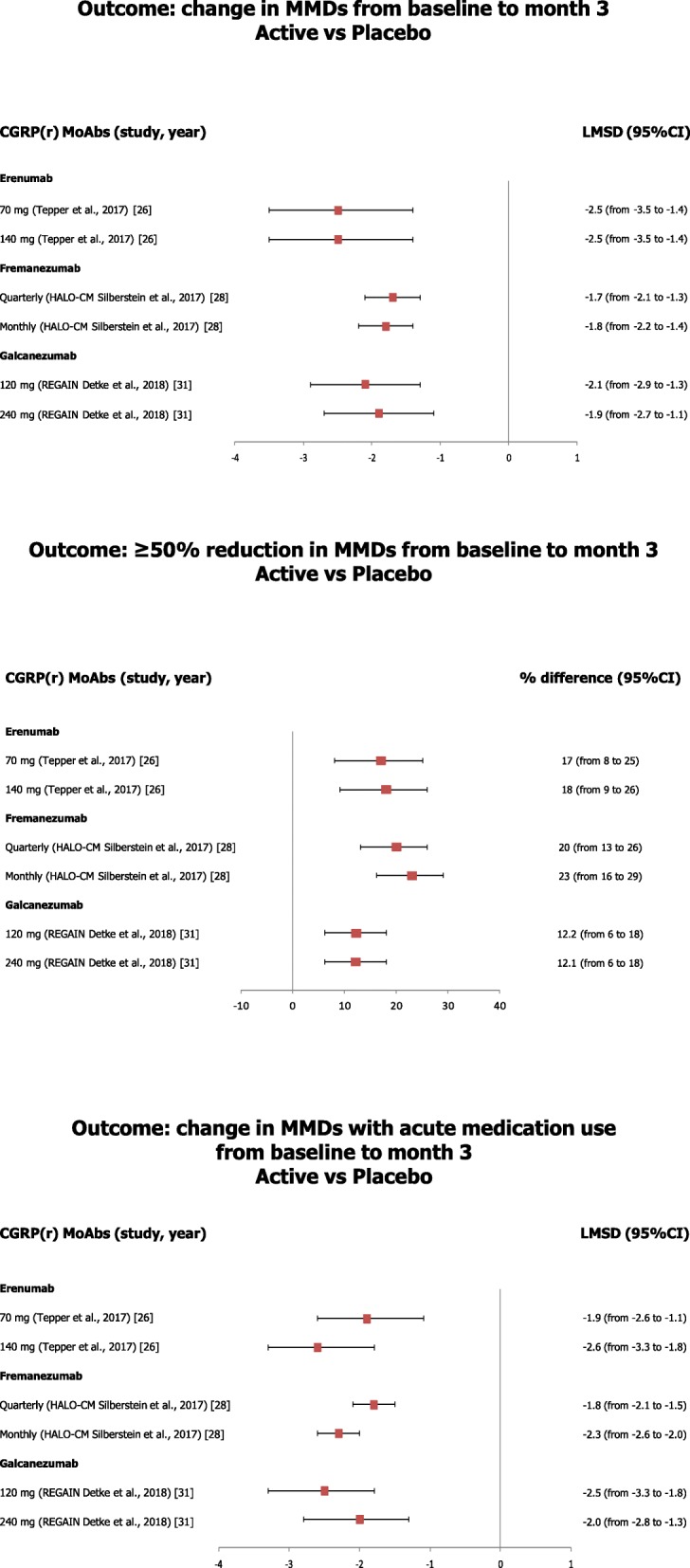
Data showing the results of CGRP(r) MoAbs on the main efficacy endpoints in patients with chronic migraine

Figures 1 and 2 provide an overview of the results on the main efficacy endpoints of CGRP(r) MoAbs observed in RCTs. In patients with EM (Fig. 1), erenumab, fremanezumab, and galcanezumab determined a modest but significant decrease, ranging from 1 to 2 days, in the mean number of MMD, a significant reduction in the monthly acute medication use and a meaningful improvement in function with respect to placebo after 12–24 weeks of treatment. Treatment with eptinezumab was superior to placebo in the reduction of the number of MMD at weeks 5–8, but a claimed effect was not confirmed at week 12; besides, eptinezumab 1000 mg determined a reduction of monthly acute medication use, but a non-significant improvement in function compared with placebo [18]. Nevertheless, preliminary results of the phase III PROMISE-1 RCT [19] showed that treatment with eptinezumab determined a significant reduction of MMD and a higher proportion of patients achieving ≥50% reduction of MMD with respect to placebo. A further clinically meaningful finding reported by RCTs is the evidence of a higher significant proportion of patients that at weeks 12–24 achieved at least 50% reduction of MMD with CGRP(r) MoAbs (from 40% to 62%) than with placebo (from 27% to 39%) [18, 24, 25, 27, 29, 30]. Notably, a proportion of patients with EM treated with CGRP(r) MoAbs had a complete response, i.e. no migraine episodes; at week 12 the proportion of complete responses was 16% for eptinezumab [18] and 35.5% for galcanezumab 120 mg [32], while at week 24 the proportion ranged from 11.5% to 15.6% for galcanezumab 120 mg, and from 13.8% to 14.6% for galcanezumab 240 mg [29, 30].

In patients with CM (Fig. 2), a 12-week treatment with erenumab, fremanezumab, and galcanezumab was associated with a significant decrease, ranging from 2 to 3 days, in the mean number of MMD [24, 26, 29], a higher rate of patients with at least 50% reduction of MMD [26, 28, 31], a significant reduction, ranging from 2 to 3 days, in the monthly acute medication use [26, 28, 31], and a meaningful improvement in function compared with placebo [28, 31]. Preliminary results of the phase III PROMISE-2 RCT [22, 23] showed that treatment with eptinezumab was associated with a significant reduction of MMD (mean MMD reduction: eptinezumab 100 mg, − 7.7; eptinezumab 300 mg, − 8.2; placebo, − 5.6) and with a higher proportion of patients achieving at least 50% reduction in MMDs (eptinezumab 100 mg, 57.6%; eptinezumab 300 mg, 61.4%; placebo, 39.3%) compared with placebo from baseline to week 12.

CGRP(r) MoAbs have shown superiority even in reducing the burden of non-head-pain symptoms of migraine, including nausea or vomiting, photophobia, and phonophobia. Indeed, post-hoc analyses of the phase II [33], of the HALO-EM [34] and HALO-CM trials [35] on fremanezumab, and of the EVOLVE-1, EVOLVE-2 and REGAIN RCTs [36] on galcanezumab showed that patients in the treatment group yielded a modest but significantly greater reduction in the mean number of days with nausea or vomiting and with phonophobia and photophobia compared with placebo from baseline to week 12 in both EM and CM patients. Moreover, evidence from preliminary data on galcanezumab suggest the possible benefit of CGRP(r) MoAbs on reducing MMD with prodromal symptoms in both patients with EM and CM [36].

Overall, data on the efficacy of CGRP(r) MoAbs on migraine associated symptoms seem to suggest that their effect goes beyond the mere control of the pain and that is possibly implicated in preventing the activation of those mechanisms leading to the complete clinical manifestation of the migraine attack. Less clear is the role of CGRP(r) MoAbs on aura symptoms; preliminary data on galcanezumab have shown the superiority of treatment compared with placebo in reducing MMD with aura in EM, but not in CM patients [36]. It would be interesting to understand whether CGRP(r) MoAbs, which cross the intact blood-brain barrier only in small amounts, might indirectly inhibit cortical spreading depression [37].

## Dose and route of administration

CGRP(r) MoAbs are administered with subcutaneous or intravenous injections. Their large dimensions, the relatively low permeability through cell membranes and instability in the gastrointestinal tract make CGRP(r) MoAbs unsuitable for oral administration. All CGRP(r) MoAbs have a long half-life in blood and a corresponding long duration of action, thus allowing for long administration intervals with monthly or quarterly dose. Moreover, none of these agents requires dose titration.

Currently, erenumab, fremanezumab, and galcanezumab have been approved at the doses of 70 mg, 225 mg, and 120 mg respectively. Table 1 summarizes the recommended dose regimens for the approved CGRP(r) MoAbs. Erenumab, fremanezumab and galcanezumab are all available for self-administration with a subcutaneous single-dose prefilled pen. The suggested sites of subcutaneous injection are upper arms, lower abdomen/belly/waistline, and front of thighs. For higher treatment dose regimen multiple consecutive injections of a single-dose might be required; the same body site can be used, but it is recommended not to inject the exact location of the first one. It would be reasonable that physicians administer the first treatment dose, to provide proper training to patients or caregivers and to evaluate possible allergic reactions.

**Table 1:** Recommended dose regimens of CGRP MoAbs for migraine prevention

Drug	Route of administration	Dosage forms	Loading dose	Recommended dose regimen
Erenumab	sc injection	70 mg/mL solution	Not required	70 mg monthly^a^
Fremanezumab	sc injection	225 mg/1.5 mL solution	Not required	225 mg monthly
			Not required	675 mg quarterly^b^
Galcanezumab	sc injection	120 mg/mL solution	240 mg^c^	120 mg monthly

^a^Two consecutive injections of 70 mg each may be reasonable for selected patients (see text)

^b^Three consecutive injections of 225 mg each

^c^Two consecutive injections of 120 mg each

## Patients eligible for treatment

As for other preventive treatments, before prescribing an CGRP(r) MoAb preliminary considerations on migraine and patient’s characteristics are mandatory. Patients to be considered for the treatment with CGRP(r) MoAbs should suffer from migraine with or without aura according to the criteria of the International Classification of Headache Disorders [38] and have at least 4 MMDs. In RCTs, CGRP(r) MoAbs have been tested in patients with EM with migraine attack frequency of at least 4 MMDs, in those with high frequency EM with at least 8 MMDs, and in those with CM with at least 15 monthly headache days, including at least 8 MMDs.

Both male and female patients aged from 18 to 70 years may benefit from treatment with CGRP(r) MoAbs for migraine prophylaxis. We have no data about the safety and efficacy of those agents in patients younger than 18 years and older than 70 years, because they were not included in RCTs. It reasonable to think that the patient’s eligibility to CGRP(r) MoAbs should go beyond age restrictions and that more important is the assessment of intercurrent clinical conditions that would contraindicate their use.

No head-to-head studies comparing CGRP(r) MoAbs with other preventive treatments are available allowing comparisons in term of efficacy [39] but the tolerability profile appears much better than that of the available drugs (Table 2). This is a very relevant point considering that in migraine, preventive treatment is mainly aimed to improve quality of life and that in many cases side effects overcome benefits related to prevention of attacks. Additionally, the infrequent administration, the lack of titration, the quick onset of action, and of the absence of interactions with other drugs, make the treatment with the CGRP(r) MoAbs appealing for physicians and patients. The main limitation of CGRP(r) MoAbs is represented by high costs which cannot make those drugs the first-line treatment for migraine prevention. In the clinical setting, it is reasonable to use CGRP(r) MoAbs in patients who failed treatment with at least two preventive drug classes because of lack of efficacy or intolerable side effects. For patients with CM, it might be reasonable to require failure of an adequate trial with onabotulinumtoxinA before starting the CGRP MoAbs. Moreover, some patients may have contraindications to oral preventive drugs further limiting the possibilities of treatment. In patients who are overweight and obese and have comorbid depression most of the available preventive drugs are contraindicated; in this group of patients, CGRP(r) MoAbs may represent the first-line treatment together with onabotulinumtoxinA if they have CM.

**Table 2:** Practical issues about CGRP(r) MoAbs versus other migraine preventive treatments with established efficacy

	CGRP(r) MoAbs	Antiepileptics	β-blockers	OnabotulinumtoxinA
Route of administration	Intravenous or subcutaneous injection	Oral	Oral	Intramuscular
Dosing frequency	Monthly or quarterly	≥1 daily	≥ 1 daily	Quarterly
Adverse effects	Rare	Common	Infrequent	Rare
Pregnancy and nursing	Contraindicated	Contraindicated	Possible with caution in the first trimester of pregnancy Not recommended during breastfeeding	Contraindicated
Costs	High	Low	Very low	Medium

*CGRP(r)* indicates Calcitonin gene-related peptide (receptor), *MoAbs* Monoclonal antibodies

## Screening before initiating CGRP(r) MoAbs

Initiation of CGRP(r) MoAbs should be preceded by a detailed history and a complete physical examination in order to evaluate the patient eligibility and to exclude possible contraindications to their use. Special attention should be paid to childbearing potential women since there are no adequate data on the developmental risk associated with the use of CGRP(r) MoAbs during pregnancy. Women of childbearing age should receive counseling on the appropriate use of methods of birth control. As a consequence of long half-life, it would be also reasonable to delay pregnancy after the last dose of CGRP(r) MoAbs. Moreover, there are no data on the presence of CGRP(r) MoAbs in human milk, therefore the effects on the breastfed infant is unknown. When evaluating to treat with CGRP(r) MoAbs women during lactation, the developmental and health benefits of breastfeeding should be considered along with the mother’s clinical need for migraine specific preventive treatment.

Patients should be also screened for preexisting cardiovascular disease before initiating treatment with CGRP(r) MoAbs. Since CGRP has a potent vasodilatator effect throughout the vascular system [40], the chronic inhibition of CGRP pathway with MoAbs and has been considered dangerous because potentially able to induce hypertension and facilitate the precipitation of coronary or cerebral hypoperfusion into ischemia [41, 42]. For these reasons, patients with cardio- and cerebrovascular diseases including history of myocardial infarction, stroke, transient ischemic attack, unstable angina, or coronary artery bypass surgery or other revascularization procedures were excluded from available RCTs [18, 24–32]. It would be reasonable to exclude from treatment patients with comorbid cardiovascular diseases, at least as long as further studies and real world registries record the long-term effects of continuous blockade of the CGRP pathway. However, results from a placebo-controlled study in a high-risk population of patients with stable angina with a median age of 65 years, the inhibition of the CGRP receptor with erenumab did not adversely affect total exercise time in a treadmill test [43]. Moreover, erenumab showed to have no relevant effect on blood pressure and on diurnal rhythm of blood pressure in patients with migraine over a 12 weeks treatment period compared with placebo [44]. Screening for psychiatric conditions, including major depression, suicide ideation, schizophrenia and bipolar disorders, and for alcohol and drug abuse should be required. It would be reasonable to treat with caution these subgroups of patients, as they have been excluded from RCTs. Nevertheless, a subgroup analysis of phase III data in EM patients showed that compared with placebo, erenumab proved effective against migraine in patients with and without depression or anxiety history [45]. Similarly, fremanezumab demonstrated efficacy in preventive treatment of CM in patients with depressive symptoms [46].

Before initiating treatment with CGRP(r) MoAbs, no specific laboratory tests are required. MoAbs are proteins degraded by tissue into endogenous amino acids with consequent fewer safety concerns and low risk of drug-drug interaction [47]. However, it is worth considering that patients with significant laboratory abnormality, hepatic disease, abnormal liver and kidney function were excluded from RCTs [18, 24–32].

## Special consideration for management of CGRP(r) MoAbs treatment

### Previous preventive treatment failures

CGRP(r) MoAbs have been tested in preventive treatment-naïve patients as well as in those who had an inadequate response or intolerance to one or more preventive treatment. Available studies in EM allowed the inclusion of patients with failure of up to 2 preventive drugs and studies in CM allowed the inclusion of patients with failure of up to 3 preventive drugs. Only the phase II RCT on eptinezumab in EM did not exclude patients according to the number of previous failure of preventive drugs [18]. Available evidence on erenumab [48, 49], fremanezumab [50, 51] and galcanezumab [52] suggests that CGRP(r) MoAbs are effective even in EM and CM patients with previous preventive treatment failures.

Evidences from RCTs on erenumab suggest that in patients with EM and at least 1 previous preventive failure both 70 and 140 mg regimens are effective, while in those with at least 2 preventive failures the efficacy of erenumab 140 mg was superior compared with placebo in reducing MMD, whereas it was only marginal with erenumab 70 mg [48]. The phase IIIb LIBERTY trial [53] evaluating the efficacy of erenumab 140 mg in preventing EM in patients with 2-to-4 preventive treatment failures confirmed the superiority of erenumab compared with placebo in the main efficacy endpoints [53]. Similarly, in patients with CM and previous preventive failure both doses of erenumab were effective compared with placebo in the main efficacy endpoints at month 3 and were greater in the subgroups of patients who have failed ≥1 or ≥ 2 prior preventive treatments than in patients with no prior treatment failure, with the greatest differences in patients who failed ≥2 prior preventive medications and treated with erenumab 140 mg [49]. Contrariwise, in patients with CM and at least 2 preventive failures, galcanezumab 120 mg was more effective than galcanezumab 240 mg in reducing MMD [52].

OnabotulinumtoxinA injections for migraine prevention were prohibited during the RCTs and for at least 4–6 months before the start of the baseline phase [26, 28, 31]. Patients who had previous use of onabotulinumtoxinA were included in RCTs but no information referring to previous efficacy of onabotulinumtoxinA and response to study treatment is available. Post-hoc analyses of phase III RCTs on galcanezumab showed that treatment significantly decreased MMD and acute migraine->specific medication use, and improved quality of life in patients who failed prior onabotulinumtoxinA treatment due to lack of efficacy or tolerability [54]. In these subgroups of patients, the greater reduction in MMD was observed with galcanezumab 240 mg with respect to galcanezumab 120 mg, while the improvement in acute migraine-specific medication use and in quality of life was similar between the two dose regimens [54].

The results of those studies suggest that CGRP(r) MoAbs represent a valid treatment option for patients with EM and CM with previous preventive treatment failure, but also for those difficult-to-treat migraine patients in whom multiple traditional oral migraine preventive treatments were unsuccessful, not tolerated, or contraindicated. Erenumab, fremanezumab, and galcanezumab were not evaluated in patients with CM refractory to current available medical treatments. However, due to the poor quality of life of patients with refractory CM it is reasonable to treat them in daily clinical practice with erenumab, fremanezumab, or galcanezumab.

### Medication overuse

Some of the available evidence indicates that CM patients with medication overuse (MO) should be withdrawn before offering preventive medications, but precise indications on detoxification strategies are not provided [55]. RCTs on erenumab, fremanezumab and galcanezumab enrolled a consistent proportion of patients with MO [26, 28, 31]. In all those RCTs patients with MO were not treated with detoxification strategies before the administration of CGRP(r) MoAbs. Preliminary data of a post-hoc analysis of the phase III HALO-CM trial [56] indicates that fremanezumab at monthly and quarterly dosing are both efficacious in the reduction of MMD in patients with CM and MO [56]. Moreover, among CM patients with baseline MO, the proportion of those reporting no MO during the 12-week treatment period was significantly higher in the quarterly (55%) and monthly (61%) fremanezumab group compared with placebo (46%). Among those patients who reverted to no MO, the monthly average number of days with acute headache medication use significantly decreased with both quarterly and monthly fremanezumab compared with placebo [56]. Therefore, it might be reasonable to offer treatment with CGRP(r) MoAbs to patients with CM and MO. In order to have a clearer picture of the migraine impact and on the effect of CGRP on migraine relief, it would be preferable to detoxify first with later or concomitant initiation of CGRP(r) MoAbs. Anyway, treatment with CGRP(r) MoAbs can be initiated even if detoxification is not feasible, contributing itself to the reduction of the number of acute headache medication.

### Association with other concomitant migraine preventive treatments

Available evidence suggests that CGRP(r) MoAbs are effective and well-tolerated even when added to other concomitant migraine preventive treatments [57, 58]. These results plausibly outline the absence of drug-drug interactions, specificity and safety of CGRP(r) MoAbs.

The add-on strategies might be especially useful in patients who experience an insufficient response to a single preventive treatment. Indeed, in patients who achieved a meaningful efficacy with a preventive medication, but migraine attack frequency or severity still produces too much disability, it would be reasonable not to stop the ongoing treatment and consider adding CGRP(r) MoAbs. Also patients that need to discontinue the preventive treatment due to the lack of efficacy or side effect would benefit from the add-on strategy with CGRP(r) MoAbs during the slow tapering phase, in order to avoid possible rebound effects.

Data from fremanezumab suggest that CM patients with concomitant use of preventive medication would benefit more from the monthly than from the quarterly dose regimen; indeed, compared with placebo, fremanezumab monthly but not fremanezumab quarterly determined a significant reduction of the mean number of monthly headache days of at least moderate severity from baseline to week 12 [58]. Nevertheless, further evidence is needed in order to elucidate the appropriate management of CGRP(r) MoAbs treatment in add-on. It is worthy to consider that all RCTs, in order to avoid confounders, did not include among the allowed concomitant preventive treatments, the use of onabotulinumtoxinA for migraine or for any medical or cosmetic reasons requiring injections in the head, face, or neck. Therefore, at the moment, the association of onabotulinumtoxinA with CGRP(r) MoAbs is not supported by evidence. As the mechanism of action of onabotulinumtoxinA is not entirely clear, it cannot be speculated whether the mechanisms of action may be synergic. From the theoretical point of view, no interaction leading to adverse events can be expected, and adding onabotulinumtoxinA and CGRP(r) MoAbs may represent a treatment strategy for the difficult-to-treat patients.

## Evaluation and management of response to treatment

### Assessment of response to treatment

The response to CGRP(r) MoAbs, as for all other preventive treatments, should be regularly monitored with pre-established time intervals in order to determine whether meaningful change is occurred and guide the decision-making process. Scheduled follow-up visits every 3 months for patients receiving monthly dose and every 6 months for those on quarterly dose should be considered. The assessment of efficacy and tolerability of preventive treatment should be based on the patients’ subjective response and by physicians’ expert opinion guided by outcome metrics. Evidence of treatment benefits would be driven by the reduction in MMD, by the reduction of acute migraine-specific medication use, and by the subjective improvement of function compared with the pretreatment period. To monitor those outcomes, the use of a headache diary is reasonable. Validated scale, including the 6-item Headache Impact Test (HIT-6) [59], the Migraine Disability Assessment Score (MIDAS) [60], the Migraine Physical Function Impact Diary (MPFID) [61], the Migraine-Specific Quality of Life Questionnaire (MSQ) [62], and the Patient Global Impression of Severity of Illness (PGI-S) [63] are especially useful for obtaining an objective measure of change in disability in everyday activity, physical impairment, perception of severity of illness, and quality of life in migraine patients.

### Management of non-responder patients

In RCTs on CGRP(r) MoAbs, patients were considered responders if achieving at least 50% reduction in MMDs [18, 24–31]. However, in clinical practice the classification of patients as responders or non-responders is less strict. In fact, patients may give value not only to the reduction in the MMDs, but also to improvement in pain severity, duration of attacks, response to acute treatments and associated symptoms. In patients with CM, even a 30% reduction in MMD might be clinical relevant, especially if it is accompanied by improvement in pain intensity, in quality of life, or of the number of headache-free days. In clinical practice, the overall satisfaction of patients is a key parameter to assess treatment benefit.

A proper timing of clinical assessment is also important to assess possible treatment failure. The available RCTs reported that CGRP(r) MoAbs have a rapid onset of efficacy over few days or weeks; however, they also reported that the proportion of patients with at least 50% MMD reduction progressively increased from week 4 to week 8 and 12 [21, 25, 29–32, 64, 65]. A relevant proportion of patients who were not responding during the first month of treatment, started having a response with prolonged treatment [66]. In the galcanezumab RCTs on EM, at month 6, 62% of patients who were non-responders after the first month achieved at least 50% reduction in MMD and 20% achieved at least 75% reduction [66]. In patients with CM, with continuation of galcanezumab treatment, at month-3, 38% of patients who were non-responders after the first month achieved at least 30% reduction in MMDs and 13% achieved at least 50% reduction [66]. In particular, the opportunity of a clinical relevant response, defined in this post hoc analysis as at least 30% reduction in MMD in patients with CM and as at least 50% reduction in MMD in patients with EM, was greatest in patients who, during the initial 2 months of treatment, had EM with limited or modest (from at least 10% to less than 50% reduction in MMD) early improvement and for those who had CM and modest early improvement (from at least 30% to < 50% reduction in MMD) [66]. According to those post hoc data, patients who do not achieve a clinically meaningful response within the first month of treatment should not discontinue the drug. Patients that after 12–24 weeks of treatment do not have a clinical meaningful response could be considered for discontinuing treatment with CGRP(r) MoAbs.

Shift from one CGRP(r) MoAbs to another might be attempted in non-responders, even if there are no available data supporting this option. Eptinezumab, fremanezumab and galcanezumab have a similar binding action to CGRP, while erenumab targets CGRP receptor. However, since peptides other than CGRP could bind to the CGRP receptor, and CGRP could bind and activate also non- CGRP receptors, it could be interesting to speculate on the possible clinical differences between blocking the peptide or its receptor [41]. Nevertheless, currently there is no evidence suggesting that patients non-responder to MoAbs targeting the peptide would respond to the MoAb targeting the receptor or vice versa.

It has been recently recognized that CGRP receptors can trigger signalling pathways not only when activated on the cell surface, but also when internalized within endosome [67]. Since the receptors within cellular compartments could be inaccessible to current CGRP(r) MoAbs, further studies should clarify the potential influence of these new drugs on CGRP receptor signalling, trafficking, and expression, and the possible consequence of CGRP receptor regulation on the effectiveness of CGRP(r) MoAbs.

### Management of responder patients over time

Data form the open-label extension of the available RCTs showed that response to treatment with CGRP(r) MoAbs is maintained over the whole 9–12 month period; the effectiveness was paralleled by a well tolerated safety profile as shown by the low incidence of adverse events [68–72]. So, at the moment there are not concerns about loss of efficacy or need of dose augmentation with prolonged use.

Up to now, with the available oral drugs or onabotulinumtoxinA, EM is mostly managed with 3–12 months of duration treatment whereas CM is mostly managed even with short- or long-term treatment. In the absence of clear evidence, it would be anyhow reasonable to stop the treatment with CGRP(r) MoAbs in patients who achieve, after at least 3–6 months of treatment, a stable reduction of migraine attacks to less than 4 days per month. After stopping CGRP(r) MoAbs, patients should be regularly re-evaluated to verify the persistency of low frequency EM. In patients who have migraine improvement but continue to experience more than 5 days per month with migraine it is reasonable to continue treatment.

Data on galcanezumab indicated that 50% of patients who were sustained responders during the 6-month treatment period, tend to have first loss of response within 4 months from treatment withdrawal [73]. Migraine, as other chronic diseases, may require long-term or even life-long treatment which is now rarely applied because of the poor tolerability of currently available migraine preventive drugs. A short-term, cyclical treatment, may be reasonable in subjects with a relatively low frequency of attacks but may not represent the best option in patients with high-frequency EM or CM.

## Adverse events

Across all available RCTs, CGRP(r) MoAbs showed that most of the treatment-emergent adverse events were mild to moderate in severity [18, 24–32]. Adverse events leading to discontinuation were infrequent (from ≤1% to 4%) in clinical trials. The most common reported adverse event was the occurrence of injection site reactions including pain, induration, and erythema with the use of subcutaneous formulations [24–32]. Constipation and reduced tolerance to fatigue represent reasons that may lead to discontinuation of treatment in some patients. Other adverse events reported in ≥2% of patients were upper respiratory tract infection, influenza, nausea, sinusitis, nasopharyngitis, arthralgia, pruritis, back pain, muscle spasm, abdominal pain, urinary tract infection, and dizziness [24–32]. No evidence of any hepatotoxicity of CGRP(R) MoAbs was reported.

Being highly specific for CGRP or its receptor, CGRP(r) MoAbs have minimal interaction with the immune system and no immunomodulatory effect [74]. CGRP(r) MoAbs are engineered to have sequences that closely resemble (humanized MoAbs) or are identical (human MoAbs) to human immunoglobulin sequences. For this reason, the risk of immunogenicity is considered low. However, antidrug antibodies can be produced and neutralizing antibodies might interfere with the effectiveness of the drugs. Data from RCTs have shown that the occurrence of neutralizing antibodies is an infrequent event (from 0 to 3.1%) [24–26, 29–31]. No specific association between the presence of antidrug antibodies and treatment safety and efficacy has been reported. Anyhow, the clinical consequence of neutralizing and binding antibodies should be further elucidated by studies with a long-term follow-up in order to evaluate the possible association with safety concern, and with loss of efficacy and alteration in half-life of CGRP(r) MoAbs. At the moment no evidence supports the need to test antidrug antibodies in clinical practice.

CGRP is involved in physiological mechanisms in the whole body, including the maintenance of cardio- and cerebrovascular homeostasis [40, 41], the facilitation of wound healing [75], and the modulation of gastrointestinal motility [76]. Nevertheless, only little evidence on the safety of long-term blockade of the entire CGRP pathway is currently available. In particular, it is crucial the comprehension of the effect of long-term CGRP(r) MoAbs on the vascular system. Indeed, CGRP acts as a vasodilatory safeguard mechanism during cerebral and cardiac ischemia [77], prevents vasospasm and subsequent ischemia after subarachnoid hemorrhage [78], reduces the generation of hypertension [79], and prevents against heart failure via chronotropic and inotropic effects [80]. It is also to be considered that migraine, in particular migraine with aura, is associated with an increased risk of cardio- and cerebrovascular diseases; a link that appears to be mediated by specific interactions among vascular risk factors and genetic, environmental, personality and psychological factors [81–84]. Thus, it is compelling to assess whether the long-term use of CGRP(r) MoAbs determine or facilitate vascular pathologies or if other protective mechanisms come into play in maintaining vascular homeostasis.

Recent evidence revealed that CGRP enhances neovascularization and lymphangiogenesis under pathological conditions [85]. Consequently, CGRP(r) MoAbs may reduce angiogenesis and enhance lymphedema, and may have a deleterious effect on the healing of gastric ulcer and skin wound, but concurrently. On the other hand, they may inhibit tumor-associated angiogenesis providing a therapeutic strategy for cancer treatment [85].

## Conclusions

CGRP(r) MoAbs are efficacious and safe treatments for migraine prevention. Becoming familiar with the practical aspects of CGRP(r) MoAb treatment is important, as their employment in clinical practice will rapidly increase. Ease of use and lack of relevant side effects represent their main strengths making them the more appealing among the migraine preventive treatments. However, because their high costs they cannot represent the first line treatment for migraine prevention but should be reserved for patients who cannot be managed with the oral treatments or with onabotulinumtoxinA. Further large-scale studies are needed to collect long-term follow-up data in order to establish safety with the long-term use, to define and manage treatment response and non-response, and to assess treatment response in patients that were excluded from the available trials, including drug resistant patients and those with CM concurrently treated with onabotulinumtoxinA.

## Abbreviations

CGRP(r): Calcitonin gene-related peptide (receptor)
CM: Chronic migraine
EM: Episodic migraine
MMD: Monthly migraine day
MO: Medication overuse
MoAbs: Monoclonal antibodies
RCT: Randomized clinical trial
